# Disease Ecology of a Low-Virulence *Mycoplasma ovipneumoniae* Strain in a Free-Ranging Desert Bighorn Sheep Population

**DOI:** 10.3390/ani12081029

**Published:** 2022-04-14

**Authors:** Brianna M. Johnson, Janice Stroud-Settles, Annette Roug, Kezia Manlove

**Affiliations:** 1 Department of Wildland Resources and Ecology Center, Utah State University, Logan, UT 84321, USA; bmj32@nau.edu; 2 Zion National Park, SR 9, 1 Zion Park Blvd, Springdale, UT 84767, USA; janice_stroud-settles@nps.gov; 3 Utah Division of Wildlife Resources, 1594 W North Temple Avenue, Salt Lake City, UT 84116, USA; aroug@ucdavis.edu; 4 Centre for Veterinary Wildlife Research, Faculty of Veterinary Science, University of Pretoria, Soutpan Road, Onderstepoort 0110, South Africa

**Keywords:** bighorn sheep, *Mycoplasma ovipneumoniae*, disease ecology, wildlife disease, strain virulence, serology

## Abstract

**Simple Summary:**

Like many wildlife diseases, bighorn sheep pneumonia can vary in burden. Here, we report on a bighorn sheep pneumonia event that showed much lower symptom and mortality burdens than have been documented previously. We provide detailed descriptions of symptoms, diagnostic testing results, and mixing patterns throughout the population, and end by discussing mechanisms that could have generated the distinct disease ecology associated with this event.

**Abstract:**

Infectious pneumonia associated with the bacterial pathogen *Mycoplasma ovipneumoniae* is an impediment to bighorn sheep (*Ovis canadensis*) population recovery throughout western North America, yet the full range of *M. ovipneumoniae* virulence in bighorn sheep is not well-understood. Here, we present data from an *M. ovipneumoniae* introduction event in the Zion desert bighorn sheep (*Ovis canadensis nelsoni*) population in southern Utah. The ensuing disease event exhibited epidemiology distinct from what has been reported elsewhere, with virtually no mortality (0 adult mortalities among 70 animals tracked over 118 animal-years; 1 lamb mortality among 40 lambs tracked through weaning in the two summers following introduction; and lamb:ewe ratios of 34.9:100 in the year immediately after introduction and 49.4:100 in the second year after introduction). Individual-level immune responses were lower than expected, and *M. ovipneumoniae* appeared to fade out approximately 1.5 to 2 years after introduction. Several mechanisms could explain the limited burden of this *M. ovipneumoniae* event. First, most work on *M. ovipneumoniae* has centered on Rocky Mountain bighorn sheep (*O. c. candensis*), but the Zion bighorns are members of the desert subspecies (*O. c. nelsoni*). Second, the particular *M. ovipneumoniae* strain involved comes from a clade of strains associated with weaker demographic responses in other settings. Third, the substructuring of the Zion population may have made this population more resilient to disease invasion and persistence. The limited burden of the disease event on the Zion bighorn population underscores a broader point in wildlife disease ecology: that one size may not fit all events.

## 1. Introduction

Before the westward expansion of European settlers and their old-world domestic sheep and goats in the nineteenth century, bighorn sheep (*Ovis canadensis*) occupied much of the mountain and rugged desert habitat in western North America [1]. As the European population in the West increased, native sheep populations declined precipitously due to habitat fragmentation and degradation, unregulated hunting, and disease [1]. While bighorn sheep are susceptible to many different pathogens [2], *Mycoplasma ovipneumoniae* is of particular concern because of its role in polymicrobial pneumonia [3,4,5]. *M. ovipneumoniae* invades the host’s upper respiratory tract, potentially impeding the motion of the mucociliary escalator [6,7] and allowing for downward invasion of normally commensal upper respiratory microflora. Those microflora can produce a polymicrobial pneumonia in the lower respiratory tract [8], which regularly produces high levels of mortality in bighorn sheep. *M. ovipneumoniae*-associated epizootics in bighorn sheep populations frequently result in all age die-offs, and a population can suffer from years of reduced recruitment after a pathogen introduction event [9,10,11]. Domestic sheep and goats can carry *M. ovipneumoniae* asymptomatically, and both of these species, along with other bighorns, can transmit the pathogen to naive bighorn sheep populations [12,13]. Following introduction, chronic carriage by adult bighorns can lead to annual pulses of pneumonia among susceptible juveniles [14], producing extended periods of low recruitment that can stymie population growth [11].

*M. ovipneumoniae* is genetically diverse. Many unique strains have been identified in both free-ranging bighorn sheep populations and domestic sheep flocks [15]. However, bighorns show little evidence of cross-strain immunity [16]. Outbreak severity varies among populations and disease events [10,17], but the mechanisms underlying variation in virulence are currently unknown. In particular, the demographic consequences and diagnostic signatures associated with low-virulence strains remain underexplored, due in part to the difficulties associated with identifying low-impact *M. ovipneumoniae* introductions.

Here, we document the natural history of a low-virulence *M. ovipneumoniae* strain introduced into the Zion bighorn population, a free-ranging desert bighorn sheep (*Ovis canadensis nelsoni*) population in southern Utah, in the summer of 2018. We compare demographic, symptom, and diagnostic patterns through two lamb-rearing seasons following this event to parallel patterns from more severe *M. ovipneumoniae*-related disease events documented elsewhere. We analyze spatial and temporal patterns of respiratory symptoms, pathogen persistence, and antibody expression throughout the population. Taken together, our analyses detail the disease dynamics of a low-virulence pathogen strain, thereby broadening our perspective on how *M. ovipneumoniae*-related disease events can manifest in bighorn populations.

## 2. Materials and Methods

### 2.1. Study Area

**Figure 1:**
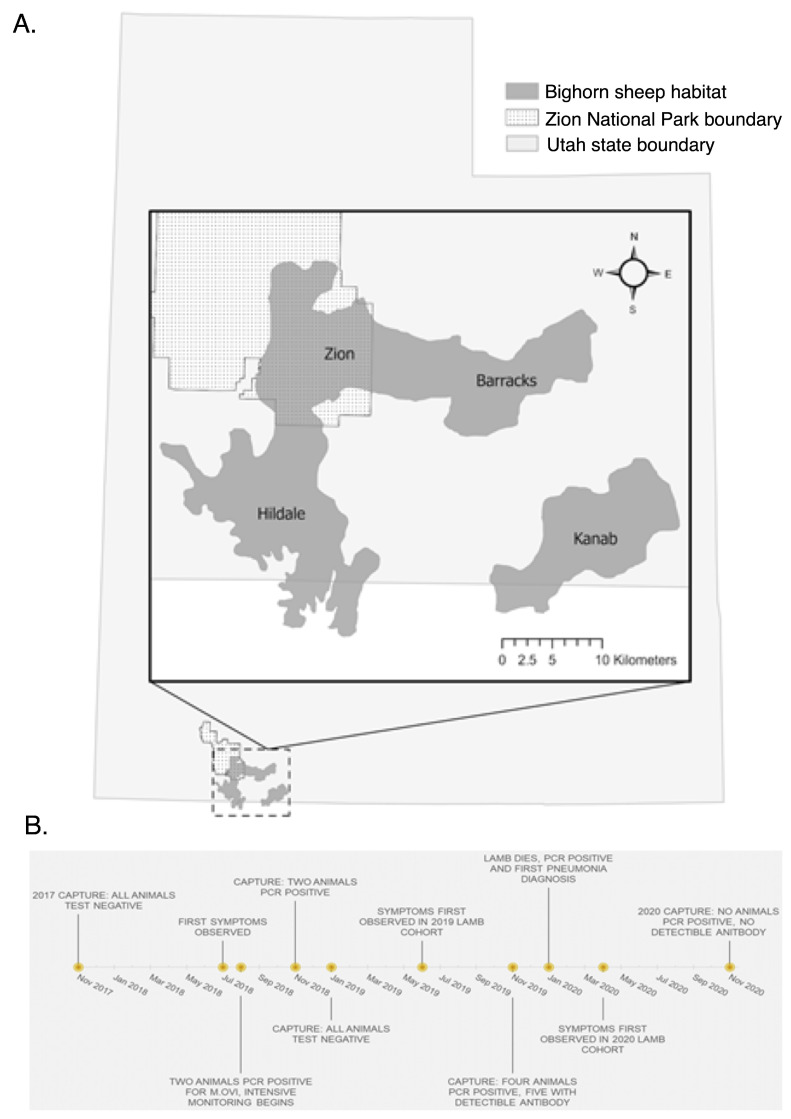
(**A**) Map of Zion bighorn habitat and subunits. (**B**) Timeline of disease events in the Zion population.

The Zion bighorn sheep population lives in rugged and variable terrain in and around Zion National Park in southwest Utah, at the junction of the Great Basin and Mojave deserts and the Colorado Plateau (Figure 1A). Elevation in the study area ranges from about 1200 m to 2200 m (3950 ft to 7200 ft), and mean annual precipitation is 41 cm. Mean summer high temperature is 37 ${}^{{}^{\circ}}$C (98.6 ${}^{{}^{\circ}}$F) and mean winter low temperature is −0.5 ${}^{{}^{\circ}}$C (31.1 ${}^{{}^{\circ}}$F). The population utilizes sandstone slickrock and canyon habitat within the National Park, south across the Arizona border, and east of U.S. Route 89 [18]. The landscape is physically substructured, resulting in four distinct subpopulations: Zion National Park ("ZNP", located within the Park), the Barracks (east of Zion National Park, along the East Fork of the Virgin River), Kanab (north and west of the city of Kanab, UT), and Hildale (south of Zion National Park, including the Canaan Mountain Wilderness and Smithsonian Butte) [18]. Bighorn sheep are native to the Zion area, but the original population was extirpated by the 1950s. The population was reestablished in the 1970s via translocation from the River Mountains in southern Nevada, and a survey in November of 2018 estimated the total population size at about 800 animals [18]. There are several other desert bighorn populations nearby, including the Beaver Dam Mountains population 50 miles to the west, the Virgin River Gorge population 50 miles to the southwest, and the Kaiparowits Plateau population 40 miles to the east.

The Zion bighorn sheep population has undergone PCR and serological testing for *M. ovipneumoniae* during all capture events since 2013 (over 75 sampling events, accounting for approximately 15% of the total population). Captures were designed to obtain random samples of the population, barring sightability and access constraints, and are thus taken to be representative of infection status across the herd as a whole. Passive surveillance was conducted on known mortality events and hunter harvests over that same period of time. Neither surveillance method revealed evidence of active or past *M. ovipneumoniae* infection in any individual within the herd until 2018, including 73 animals handled in November and December of 2017.

Clinical signs of respiratory disease emerged in the Zion National Park (ZNP) unit of the Zion bighorn population during the summer of 2018, and two animals subsequently tested PCR-positive for *M. ovipneumoniae*. Active upper respiratory tract infections and lack of measurable antibody responses in both animals suggested that exposure was quite recent.

### 2.2. Data Collection

#### 2.2.1. Captures

Animals from the Zion population were sampled over the course of seven separate capture events between 2015 and 2021 (Table 1). All captures occurred between October and January, and capture seasons are labeled according to the year in which the capture began (e.g., “capture year 2015” refers to captures occurring between October 2015 and January 2016). Most captures were conducted via netgunning from a helicopter [19], and animals were released at the capture location immediately after processing. Fifty animals bound for translocation out of the Zion herd in 2017 were transported in slings suspended from the helicopter and flown to a staging area to be loaded into trailers for translocation (Table 1). An additional 121 animals were fitted with GPS collars and released back into the population. There were 178 capture events in total (four animals were handled twice). Animals that were slung all the way to a processing site were given subcutaneous injections of approximately 1 mg/kg flunixin meglumine (50 mg/mL, Merck Animal Health, Madison, New Jersey) and 900–1500 IU vitamin E-AD (VetOne, Boise, Idaho), and animals destined for translocation were tranquilized with 10–11 mg of haloperidol (Wildlife Pharmaceuticals, Laramie, Wyoming). These amounts were for adult female sheep. Helicopter captures were conducted collaboratively with Utah Division of Wildlife Resources (UDWR) personnel and personnel from Helicopter Wildlife Services, Austin, Texas. All captures were conducted in compliance with the UDWR and National Park Service (NPS) bighorn sheep capture protocols and management plan [18].

Inside ZNP, many animals were sufficiently habituated to allow for immobilization via pneumatic dart. Darted animals were anesthetized with a pre-mixed combination of butorphanol tartrate (27.3 mg/mL), azaperone tartrate (9.1 mg/mL), and medetomidine hydrochloride (10.9 mg/mL) (BAM, Wildlife Pharmaceuticals, Laramie, Wyoming): 1.3 mL for adult females and 1.6 mL for adult males. After processing, chemical immobilization was reversed using individual intramuscular injections of Tolazoline (200 mg), Atipamezole (5 mg per mg medetomidine), and Naltrexone (1 mg per mg of butorphanol).

#### 2.2.2. Demographic Monitoring

Twenty-one radiocollars were deployed in the Barracks subunit, 26 in Hildale, six in Kanab, and 68 in ZNP. Most animals carried collars for multiple years, resulting in a total of 118 tracked animal-years from 2018 through January of 2021. Observational data were collected from January, 2019 through September, 2021. We located radiocollared individuals using VHF telemetry and recorded group size, group composition, behavior, and symptoms. Observation frequency was highest during the lambing and lamb-rearing season. A total of 524 individual observations were made in 2019, 329 in 2020, and 286 in 2021. Of those observations, 180 included lambs in 2019, 276 in 2020, and 178 in 2021. Intervals between consecutive lamb observation events ranged from 3 to 174 days. A ewe was classified as having a lamb if a lamb was observed following, nursing from, bedded with, or otherwise physically interacting with the ewe.

Collars were programmed to identify mortality after 6–10 h without movement, and each mortality signal during the study period was investigated to determine cause of death and conduct additional pathogen surveillance. All field data were gathered in compliance with Utah State University Institutional Animal Care and Use protocols #10146 and #11812.

#### 2.2.3. Diagnostic Testing for Serology and Pathogen Load

Disease sampling was conducted during all animal handling events, and additional opportunities for PCR testing occurred through 10 hunter harvests and 25 incidental mortalities. A pulse of sampling occurred in the fall of 2018 and winter of 2019 after the disease introduction event. Additional sampling was conducted in November and December of 2020 and sporadically in post-mortem settings following instrumented animal mortalities. A total of 209 PCR tests and 171 serological tests were performed over the course of the study. Samples collected from live animals included a nasal swab which was evaluated for live *M. ovipneumoniae* via polymerase chain reaction (PCR), and blood serum which was evaluated for antibodies to *M. ovipneumoniae* via cELISA. Primers for the PCR are reported in [13,16], and protocols for the cELISA are reported in [20]. Post-mortem sampling included nasal swabs and pericardial blood, and fresh and formalin-fixed tissue samples whenever possible. All diagnostic tests and pathological examinations were performed by the Washington Animal Disease and Diagnostic Laboratory (WADDL) in Pullman, Washington.

#### 2.2.4. Symptom Emergence and Intensity

We tracked the timing and severity of symptoms in lambs through the spring and summer of 2019 and 2020 using methods adapted from published protocols for documenting symptom emergence in bighorn sheep [16,21,22]. We located marked ewes using very high frequency (VHF) telemetry and observed animals through binoculars or a spotting scope. Our minimum observation duration was 60 min, and our median inter-observation interval for specific animals was 26 days (range: 3–174 days). Animals were evaluated for visible symptoms of pneumonia, including coughing, sneezing, head-shaking, nasal discharge, drooping ears, and lethargy [21], on every observation event. Each time the lamb of a known radiocollared ewe was located, the observation was assigned a score between zero and five based on the sum of scores associated with all symptoms observed. Nasal discharge contributed one point to the total score if present; head shaking, droopy ears, and lethargy contributed half a point each. Individual coughing bouts were counted throughout an observation. Zero points were assigned in the absence of any coughs; one point was assigned for less than five coughs; two points were assigned for five-to-ten coughs; and three points were assigned for more than 10 coughs. To describe symptom emergence in adults, the same clinical signs were recorded, but scores were aggregated across individuals in a group, and the resulting score was divided by the number of adult animals in the group.

### 2.3. Data Analysis

#### 2.3.1. Adult and Juvenile Survival

We constructed Kaplan–Meier estimators to explore survival patterns separately in lambs and adults with the survfit function in the survival package in R [23]. Survival across groups and years was compared using nonparametric log-rank tests implemented through the function survdiff in the same package. Adults were left-censored at their date of capture, and departed the study through mortalities, collar drop-offs, or collar failures. Departure date was identified as the earliest location in a point cluster during which the collar went into mortality mode (triggered by six to ten consecutive hours without movement for natural mortalities), the date of drop-off for a collar that was released during the study, or December 31, 2020 for all other animals. We coded collar failure (*n* = 6 animals), scheduled or accidental collar drop-off (*n* = 15), harvest (*n* = 2), and survival through the end of the study year (*n* = 65) as “censoring” events. Analyses were conducted on a calendar-year timescale [24], meaning that we tracked survival over calendar days within the year, as opposed to animal ages. Each new year in which the animal was alive and collared in the study contributed a new sampling unit.

Each ewe’s lamb entered the study on the midpoint between the last day the ewe was seen without a lamb and the first day the ewe was seen with a lamb. When a lamb was lost, its departure date was assigned the midpoint between the last day the lamb was seen alive and the first day the ewe was observed without a lamb. All lambs were censored after 180 days of age (which we regarded as the approximate age of weaning), or earlier if their dams died prior to weaning.

We calculated lamb:ewe ratios using all field observations gathered after 1 September in 2019 and 2020, and generated uncertainty estimates for those ratios by building bootstrapped confidence intervals in which groups observed after 1 September within each year were resampled with replacement 1000 times. Lamb:ewe ratios were recalculated upon each resampling, and the 2.5th and 97.5th quantiles of the bootstrapped distributions were extracted and treated as interval bounds.

#### 2.3.2. *M. ovipneumoniae* Severity and Spatial Distribution of Disease

We used symptom scores along with diagnostic data to compare virulence of the *M. ovipneumoniae* strain found at Zion to virulence of other *M. ovipneumoniae* strains in two ways: first, by modeling the rate and pattern of symptom emergence in lambs and adults as a function of the infecting strain; and second, by comparing *M. ovipneumoniae* antibody expression in the Zion population across years to serological patterns documented during disease events in other systems.

Additionally, we examined spatial patterns of disease throughout the Zion population by aggregating global positioning system (GPS) locations from collared animals and constructing spatial networks at the population and subunit levels. These were individual association networks, in which nodes corresponded to individuals and edges were weighted according to the frequency of GPS locations within 200 m on a given day. Each node in the network included demographic, reproductive, and symptom information. We described both the within-subunit and among-subunit networks in terms of modularity, betweenness, and community assortativity.

#### 2.3.3. Symptom Emergence and Scoring

We compared the timing of symptom emergence among lambs at Zion to symptom timing data from another well-documented *M. ovipneumoniae* introduction event at the Black Butte population in southeast Washington, USA [16]. We used a randomization test to compare the day of the year on which symptoms were observed in the Zion and Black Butte populations in the first year following *M. ovipneumoniae* introduction (2019 for Zion), taking the difference in median date of symptoms as our test statistic. We generated permuted datasets and recalculated the difference in median dates under those permutations to construct a reference distribution. We then compared the realized difference in median date of symptoms to the permutation distribution to calculate a p-value. We used the same protocol to compare symptom severity scores between the Black Butte and Zion events.

#### 2.3.4. Comparison of Serology and Pathogen Load with More Severe Disease Events

We used longitudinal sampling of the originally-exposed portion of the Zion population (and sometimes the same individuals) to characterize antibody signal strength (a potential corollary of antibody binding) through time. We contrasted the rate at which antibody signal strength increased with rates for a more virulent *M. ovipneumoniae* strain observed in detail in a captive setting (at Hardware Ranch, UT; Manlove et al. in revision), and another well-documented disease event in a free-ranging California bighorn sheep (*O. c. californiana*) population in northern Nevada (the Snowstorms population). To do this, we assigned a mean day of first exposure to all individuals within each population (22 February for Hardware Ranch; 15 July for Zion). We then calculated days-post-exposure for each field sample. We regressed measured percent inhibition for each animal on days post exposure. We included separate intercept terms and allowed slopes to vary among the Snowstorms, Hardware Ranch, and Zion animals. We similarly compared pathogen load (measured in PCR cycle thresholds) between the Zion, Hardware Ranch, and Snowstorms disease events.

#### 2.3.5. Social Mixing Dynamics

Social networks in which nodes represented individuals and edges represented co-occurrence of those individuals in space and time were constructed for GPS-collared animals in the Zion population. We constructed networks for single seasons, single years, and over multiple years. Association events were identified by calculating distances between pairs of animals on each day of the study and classifying animals within 200 m of one another at any point during the day as “associating”. Edge weights linking pairs of individuals were built by calculating the number of times a pair of animals was detected in association with one another, divided by the number of possible observations for that pair under an approach similar to a simple ratio index [25]. Network analyses were conducted using igraph package in R [26]. Edge weights between each pair of individuals were aggregated into networks. Following construction of each network, we used a four-step walktrap algorithm [27] to identify “communities” of individuals and then used those community assignments to determine modularity. We used the assortnet package to calculate assortativity (the extent to which nodes with similar attributes—in our case, similar symptom status—grouped together within the graph) [28]. Finally, we examined assortativity of the occurrence of respiratory symptoms to see if symptoms localized within particular regions of the network.

## 3. Results

### 3.1. Outbreak Severity and Duration

**Figure 2:**
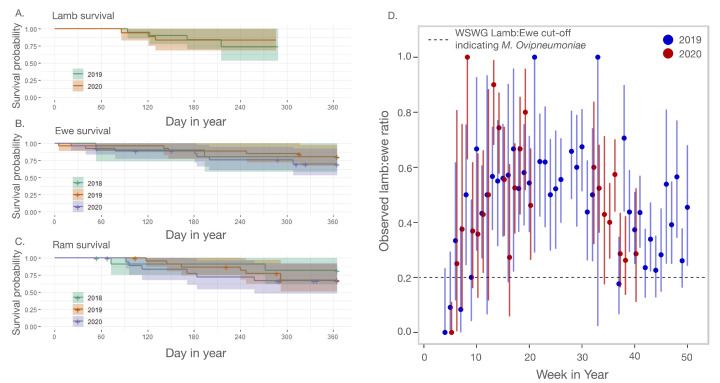
Animal survival throughout the outbreak. (**A**–**C**) Kaplan–Meier curves showing survival of lambs, ewes, and rams in the Zion population in each of the three study years. Cross-hatches indicate the dates of censoring events. Shaded regions extend to 95% confidence limits. Kaplan–Meier curves were built using the survdiff function in the survival package in R. (**D**) Weekly lamb:ewe ratios derived from field observations over the course of 2019 and 2020. Lines extend to 95% binomial confidence limits, in which the total number of trials was the total number of ewes observed over the course of the week. The dashed line shows the Wild Sheep Working Group (WSWG) cut-off for categorizing a population as experiencing problematic *M. ovipneumoniae*.

We used data from 70 radiocollared adult bighorns to assess survival before, during, and after the *M. ovipneumoniae* introduction event in summer of 2018 (Figure 2). Among these 70, 37 individuals wore collars over multiple years, resulting in 118 animal-years included in analysis: 10 ewes and 12 rams in 2018, 26 ewes and 24 rams in 2019, and 27 ewes and 19 rams in 2020. We excluded animal-years in which the collar failed (*n* = 4 animals), the collar was replaced (*n* = 3), the collar dropped off (*n* = 15), or the animal was harvested (*n* = 2), leaving 94 adult animal-years. Mortalities occurred in 29 of those animal-years: 4 in 2018, 12 in 2019, and 13 in 2020, (annual survival probability = 0.69; 95% binomial CI: [0.59, 0.78]) (Table A1). Annual adult survival probability was estimated at 0.74 within ZNP (95% binomial CI: [0.62, 0.84]). The Kanab, Barracks, and Hildale units exhibited an annual adult survival probability = 0.61 (95% binomial CI: [0.33, 0.74]). The majority of deaths were due to predation (*n* = 17) or environmental hazards, including falls and drowning (*n* = 6). The individual cause for each mortality is listed in Table A1. No adult mortalities were attributed to pneumonia, no carcasses revealed evidence of active *M. ovipneumoniae* infection based on PCR testing, and only one animal had an unknown cause of death.

We assessed summer lamb survival using data from 20 lambs born to collared ewes in both 2019 and 2020 (Table A2). Four lambs were lost in 2019, and the rest were censored if their dams died (*n* = 2), or at their approximate dates of weaning, which we took to be 180 days after entering the study (*n* = 14). Three lambs were lost in 2020, and the rest were either censored at ewe death (*n* = 3) or at weaning (*n* = 14). In 2019, 14 of the 18 tracked lambs whose dams survived also survived to weaning (95% binomial CI for recruitment probability = [0.52, 0.94]). In 2020, 14 of 17 lambs whose dams survived also survived to weaning (95% binomial CI: [0.56, 0.96]). We did not detect any difference in mortality patterns in either adults or lambs before vs. after the *M. ovipneumoniae* invasion (χ-square test statistic for adults = 1.4, *p*-value = 0.5; χ-square test statistic for lambs = 0.1, *p*-value = 0.8).

We observed 152 groups after 1 September 2019, and 87 groups after 1 September 2020. The lamb:ewe ratio calculated across all of these “late” groups in 2019 was 34.9 lambs:100 ewes (95% bootstrapped confidence interval [30.2:100, 40.5:100]). In 2020, the lamb:ewe ratio in “late” groups was 49.4 lambs:100 ewes (95% bootstrapped confidence interval [33.2:100, 77.6:100]). We tracked weekly lamb:ewe ratios over the course of both the 2019 and 2020 field seasons (Figure 2D). Ratios typically peaked in early April and then declined, but they did not drop to the 20 lamb:100 ewe level that is typically regarded as indicative of problematic disease [11].

None of the 35 animals sampled in November and December of 2020 were PCR-positive for *M. ovipneumoniae*, and only four had antibody percent inhibition levels that exceeded the WADDL cut-off for classification as seropositive, leaving us to conclude that the strain had likely faded out of the population.

### 3.2. Symptom Emergence and Intensity

**Figure 3:**
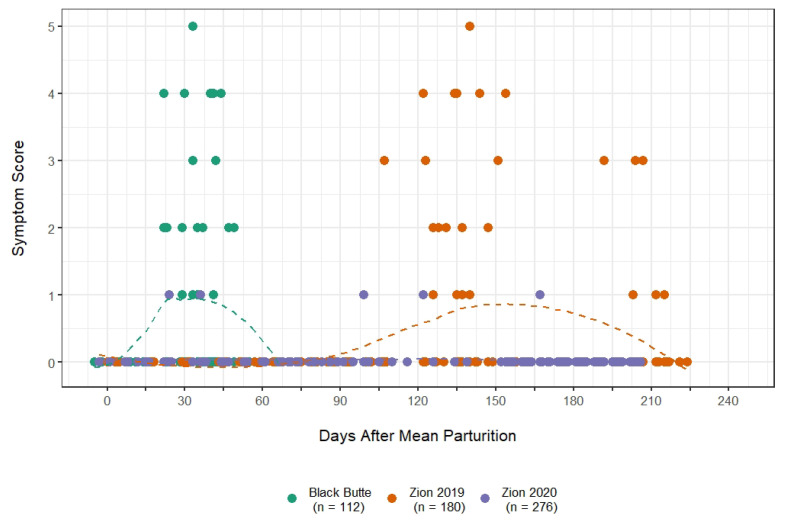
Symptom emergence patterns in Zion lambs in 2019 (orange) and 2020 (purple) in contrast to emergence in lambs from the Black Butte population (green) of Rocky Mountain bighorn sheep in 2014 (data from [16]). Lines are locally weighted least-squares lines fit to the Black Butte and Zion datasets. The same data, but aligned by day-in-year as opposed to lamb age, are presented in Figure A1.

**Figure 4:**
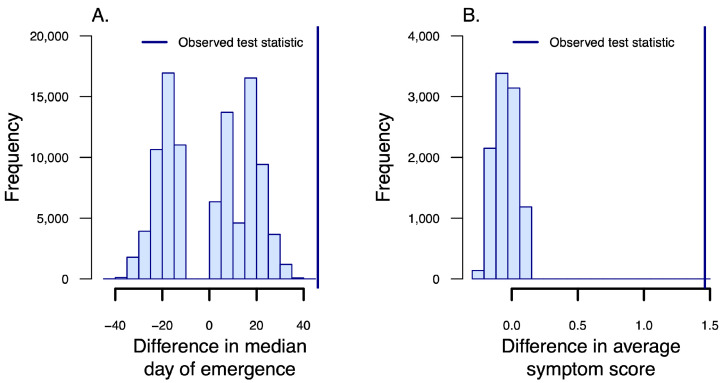
(**A**) Permutation distribution of the difference in median date of symptom emergence among Zion and Black Butte lambs in the first year following *M. ovipneumoniae* introduction. (**B**) Permutation distribution of the difference in average symptom score among Zion lambs in 2019 and 2020. The dashed vertical line shows the observed difference relative to the randomization distribution in each case.

We observed symptoms of any type (coughing, nasal discharge, ear paresis, or lethargy) in 55 of 199 lamb observations in 2019, and in 9 of 180 lamb observations in 2020. Timing and severity of symptoms among Zion lambs were distinct from what has been reported elsewhere (Figure 3), and we quantified that difference by comparing symptom data from the Zion lambs to published symptom data from lambs in the Black Butte population in Washington State. The Black Butte population was the site of an *M. ovipneumoniae* introduction event in 1995 [29], and was subject to persistent disease for many years thereafter [9,10]. In 2014, the population was estimated at approximately 45 animals, living in three relatively segregated groups [16]. Symptom records are from lambs born to one relatively closed group of 13 affected ewes that was monitored on a near-daily basis between early May and late August. Symptoms were first observed among lambs in the Zion population on June 24th, 2019. This is 22 days later than at Black Butte (symptoms first reported May 31st), despite the fact that lambs at Zion were born an average of seven weeks earlier than at Black Butte. The permutation test for symptom emergence produced a p-value of 0, suggesting that this discrepancy was unlikely to have arisen by chance alone (Figure 4A). The difference in average symptom scores in the Zion population between 2019 and 2020 was 1.46 (average in 2019 = 2.46; average in 2020 = 1.00; Figure 4B).

### 3.3. Serology and Pathogen Load

**Figure 5:**
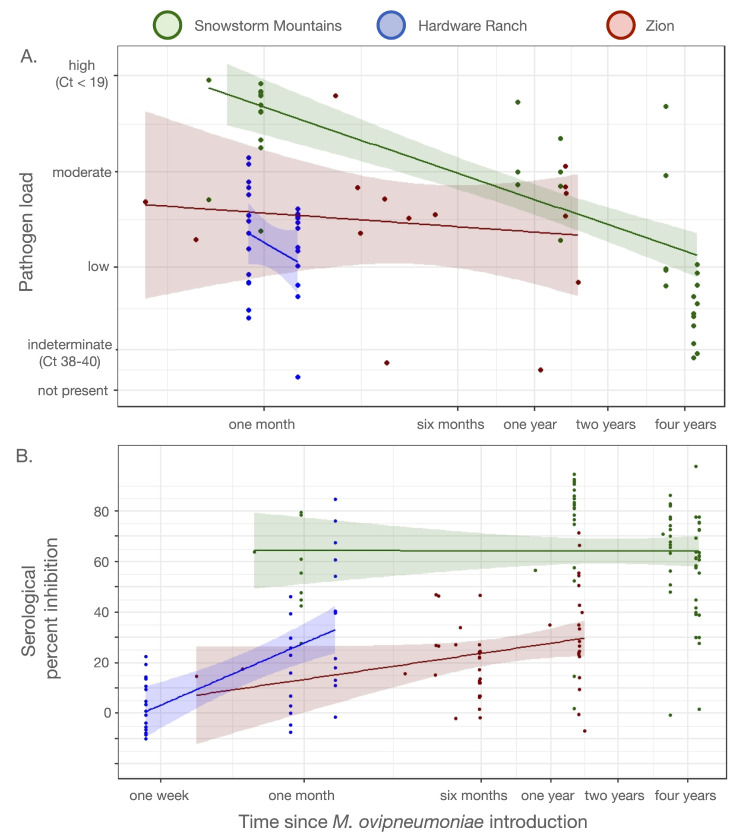
Serological and pathogen load dynamics in the Zion bighorn population (red) in contrast to dynamics from two other example populations: captive Rocky Mountain bighorn sheep at Hardware Ranch in northern Utah (blue); and free-ranging California bighorn sheep from the Snowstorm Mountains in northern Nevada (green).

Pathogen load (measured in terms of PCR cycle thresholds [Cts]) was similar to what has been observed in other comparable outbreaks (Figure 5). Antibody percent-inhibition values were substantially lower at Zion than in the two comparison events (Figure 5B), and rarely rose to the level that the Washington Animal Disease Diagnostic Lab (WADDL) uses to categorize animals as “seropositive”. This is despite the fact that although the WADDL test is primarily designed for classifying populations, not individuals, the test has a reported diagnostic specificity of 90.7% and a sensitivity of 98.7% at the individual level, according to WADDL documentation. Moreover, several animals with very low percent inhibition values were simultaneously PCR-positive. This suggests that the *M. ovipneumoniae* strain invading the Zion population did not induce a particularly powerful immune response for the antibody targeted in the cELISA test. We tentatively believe that this may be a feature of the *M. ovipneumoniae* strain, as opposed to the Zion bighorns themselves, because translocated animals from the Zion population that encountered a different *M. ovipneumoniae* strain circulating in the Dark Canyon Wilderness area north of the San Juan River following translocation showed severe disease, high mortality burdens, and strong serological signals (UDWR personal communication, 15 February 2022).

### 3.4. Social Mixing Dynamics

**Figure 6:**
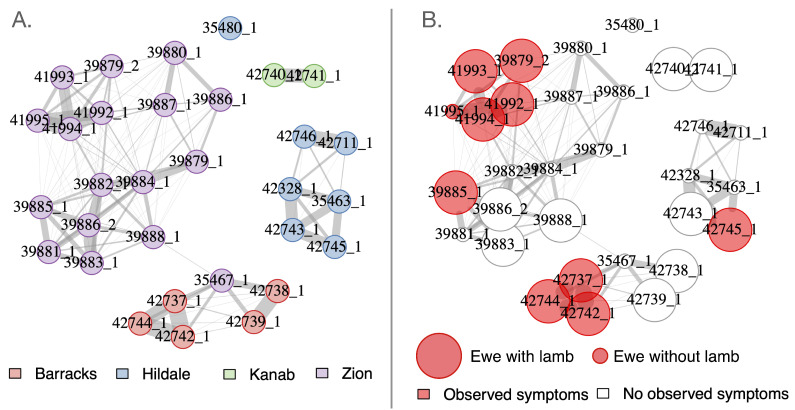
Ewe association network during the 2019 field study. The graph’s layout was determined using a Fruchterman–Reingold graph projection, which pulls nodes toward one another in proportion to the strength of association between the two individuals involved. (**A**) Social network with animal nodes colored according to their original release areas (“Zion” = within ZNP). (**B**) Social network with animal nodes colored according to whether or not they were observed in a group displaying pneumonia symptoms (red indicates animals that were observed with symptoms; white indicates animals that were never observed with symptoms).

Metrics describing the Zion social networks are presented in Table 2. The Zion population broke into four distinct communities (Figure 6A), and there was very little movement among communities by ewes (rams occasionally moved among units especially during and just prior to rut). Symptoms appeared to cluster within a few particular units, and showed evidence of a non-random distribution within the system (Figure 6B), but assortativity of symptoms was not statistically different from zero (assortativity coefficient according to symptom presence on the weighted graph = −0.03). Thus, the evidence that *M. ovipneumoniae* may have been “socially trapped” in particular ewe groups [30,31,32] remains marginal in this case.

## 4. Discussion

Here, we reported on the introduction of a novel *M. ovipneumoniae* strain into the Zion desert bighorn sheep population. The ensuing disease event produced clinical signs similar to those reported during other *M. ovipneumoniae* introductions [5,9,22,33], yet the Zion population did not exhibit a corresponding pulse of mortality (Figure 2), and the strain apparently faded out two years after introduction. Monitoring throughout 2019 and 2020 revealed symptom progression in lambs that was clinically similar, but substantially delayed, relative to patterns reported in other bighorn sheep populations (Figure 3 and Figure 4) [10,16,34].

Serological patterns deviated from those of other well-studied bighorn populations: animals produced percent inhibition values that demonstrated a longitudinal amplification pattern consistent with recent exposure, but signal strength remained substantially lower than is typical of infected populations. Over the course of the study, very few of the animals in the Zion population would have been classified as “seropositive” according to the WADDL cut-off value of 40% inhibition. The WADDL cut-off value was developed using data primarily from Rocky Mountain and California bighorn sheep. It is possible that desert bighorn sheep produce lower antibody responses, or alternatively, that this particular *M. ovipneumoniae* strain did not result in detectable responses. Misclassification of truly-exposed animals (or populations) as seronegative could have direct ramifications on disease management in this system, including whether to treat populations as translocation source populations, or how to prioritize disease vs. habitat management endeavors. Tight collaboration between intensive field investigations and the diagnostic laboratories would help to clarify the existence of subspecies-specific (or strain-specific) differences in antibody expression in the future.

### 4.1. Comparison of the Epidemiology with Other *M. ovipneumoniae* Disease Events in Bighorns

Desert bighorns can experience fairly severe all-age die-offs associated with *M. ovipneumoniae* infection (for example, this occurred in the Black Mountains of Arizona, where approximately 75% of a population was lost over a five-year interval [35]). Our survival estimates were similar to, or slightly below, post-*M. ovipneumoniae*-introduction adult survival rates reported for nine Mojave Desert bighorn populations [36], though the sources of mortality in the Zion population (predominantly predation) were distinct from those in the Mojave (where a sustained burden of *M. ovipneumoniae*-associated mortality was detected). However, very mild disease in desert bighorns is not unprecedented. For example, *M. ovipneumoniae* was present but went undetected for long periods of time in one metapopulation prior to its detection only after a novel introduction event, e.g., [37].

In the Rocky Mountain bighorn sheep subspecies, data reflecting *M. ovipneumoniae*’s associations with core vital rates are more abundant. Survival during die-offs ranged widely in one review of 82 disease events across seven states, but had a median estimated value of 48% within affected populations [17]. Lamb:ewe ratios in the presence of disease are regularly around 20 lambs:100 ewes, although one study estimated a post-introduction lamb:ewe ratio of 0.32 across 10 populations and 18 years of intensive monitoring [11]. This value is similar to what we recorded here in 2019. However, pneumonia symptoms were consistently observed in lambs throughout that system [9,10,16], whereas we saw very few symptomatic ewes or lambs in the Zion population. Moreover, severe drought in the vicinity of our study area in 2018 may have contributed to lamb survival in 2019. However, we cannot rule out the possibility that *M. ovipneumoniae*’s effects were larger than what we detected under this sampling scheme.

### 4.2. Mechanisms That Could Have Produced the Reduced Disease Burden

At least three processes could lead to the limited burden of the Zion *M. ovipneumoniae* event. On the host side, the bighorn sheep at Zion may be more genetically resilient to *M. ovipneumoniae* infection. However, we suspect this is unlikely for two reasons. First, the Zion bighorn population was established using bighorn sheep from the River Mountains in Nevada, and other “River Mountains source” populations have undergone severe pneumonia events (including an event in the River Mountains themselves; [15]). Second, animals sourced from the Zion population experienced substantial *M. ovipneumoniae*-associated pneumonia mortalities following translocation to the Dark Canyon Wilderness Area in 2017. Thirty-one radiocollars were deployed on translocated bighorns during the translocation, and 21 of those animals died between January 21, 2018 and May 2, 2021. Though specific causes of death could not be determined in many of these cases due to elapsed time between mortality and investigation or terrain access, very few showed evidence of mountain lion (*Puma concolor*) predation, and most were simply skeletons lying on slickrock or near water sources. A subset of the surviving animals were captured and tested in January of 2020, and by that time, four of the original Zion animals had seroconverted to be *M. ovipneumoniae*-positive. While other factors, including recent translocation, could have contributed to the animals’ susceptibility to infection, it is perhaps more parsimonious to conclude that host genetics are likely not the fundamental factor that reduced disease burden at Zion.

Second, the environmental context surrounding the Zion disease event could have limited disease burden in some way [38]. Plausible mechanisms here could be: (1) a diffuse birth pulse limiting the potential for severe transmission and generation of new chronic carriers in spring (or generally lower rates of lamb-to-lamb contacts); (2) better animal condition could have improved animals’ abilities to ward off infection; or (3) a more limited pathogen community beyond *M. ovipneumoniae* could have limited overall disease burden [39]. However, while the Zion birth pulse is more diffuse than those of the Rocky Mountain and California bighorns that have been targets for the preponderance of *M. ovipneumoniae* research, the birth pulse at Zion is still substantially more compact than in many desert bighorn populations (the birth pulse within the Park is approximately three months in duration, in contrast to over seven months reported in some harsh desert environments [40]. Moreover, even populations with very diffuse birth pulses can still experience severe *M. ovipneumoniae*-associated mortalities [36]. We have limited direct physiological information on the condition of the Zion bighorn sheep (ground darting in complex terrain by multiple teams precluded use of an ultrasound, and we did not gather blood for gene transcription profiles during the study). Whether the animals at Zion are in better condition than the typical desert bighorn sheep is difficult to say, but we note that *M. ovipneumoniae* apparently faded out in one of the most severe drought years in recorded history. Finally, aerobic cultures revealed that members of the Zion bighorn population harbored an array of microorganisms regularly associated with the bighorn sheep pneumonia complex, including leukotoxin-positive Pasteurellas, *Mannheimia haemolytica*, and *Bibersteinia trehalosi*, throughout the disease event.

A third option is that the particular *M. ovipneumoniae* strain that infected the Zion bighorn population may be less virulent than strains involved in other well-studied disease events. The infecting strain here is closely related to a clade of strains that may be associated with lower-burden disease events [15] (though hard evidence for this is limited, since these strains predominantly circulate in the eastern Mojave Desert, where environmental variation can lead to dramatic variation in lamb survival even in the absence of infectious disease). At present, we speculate that strain virulence, perhaps in interaction with some attribute of host physiology, is what led to the lower disease burden associated with the event described here.

Regardless of the cause, the disease dynamics reported here are measurably different from what has been documented during other, higher-virulence disease events in bighorn sheep [16,17], in terms of both the rate at which antibody signal strength increased and the apparent force of infection. Ecological and behavioral differences between the Zion bighorn population and other well-studied bighorn populations, and in particular, disparities in birth pulse timing and patterns of group mixing dynamics, may have also contributed to differences in symptom progression following pathogen introduction. Documenting heterogeneity in disease outcome is a critical first step toward understanding its sources and potentially improving our ability to understand and respond to varying disease risk in this conservation-relevant wildlife disease system.

## Figures and Tables

**Table 1:** Summary of animal handling and diagnostic testing in the Zion bighorn population, 2015–2020. We followed the standard WADDL cut-off, and categorized animals as “seropositive” if their cELISA percent inhibition was about 40% (for an indeterminate) or 50% (for a fully positive animal). * indicates animals that were tested following capture within the Zion population, but that were subsequently translocated out of the population.

Subunit & Season	Dates	Total Sampled	Ewes	Rams	Juv.	Collars Deployed	PCR-pos. (Total Tests)	cELISA-pos., indeterm. (Total Tests)	Agency	Method
Barracks 2015	31 October 2015	6	6	0	0	6	0 (6)	0, 0 (6)	UDWR	helicopter
Hildale 2015	30–31 October 2015	10	10	0	0	10	0 (10)	0, 0 (10)	UDWR	helicopter
ZNP 2017	15 November 2017 and 4 December 2017	23	10	13	0	23	0 (23)	0, 0 (23)	NPS & UDWR	helicopter
ZNP 2017	12–13 December 2017	50 *	41 *	5 *	4	0	0 (50)	0, 0 (49)	NPS & UDWR	helicopter
Barracks 2018–19	9 January 2019	9	5	4	0	9	0 (9)	0, 1 (9)	UDWR	helicopter
Hildale 2018–19	9 January 2019	9	5	4	0	9	0 (9)	0, 0 (9)	UDWR	helicopter
Kanab 2018–19	8 January 2019	4	2	2	0	4	0 (4)	0, 0 (4)	UDWR	helicopter
ZNP 2018–19	5 November–1 December 2018	11	6	5	0	11	2 (10)	0, 2 (10)	NPS & UDWR	ground
ZNP 2019–20	7 November 2018–30 January 2019	21	7	8	6	15	4 (18)	5, 1 (18)	NPS & UDWR	ground
Barracks 2020	13 December 2020	6	4	2	0	6	0 (6)	2,0 (6)	UDWR	helicopter
Hildale 2020	11 December 2020	7	5	2	0	7	0 (7)	0, 1 (7)	UDWR	helicopter
Kanab 2020	13 December 2020	2	1	1	0	2	0 (2)	0, 0 (2)	NPS & UDWR	group & helicopter
ZNP 2020	6 November–12 December 2020	20	17	3	0	19	0 (20)	1, 0 (18)	NPS & UDWR	ground & helicopter

**Table 2:** Network metrics associated with various partitions of the Zion bighorn population data.

Level	Communities	Assortativity	Assort. *p*-Value	Betweenness	Between. *p*-Value	Modularity
Whole population all years	16	0.16	0.007	666	0.1896	0.16
Ewes all years	8	0.21	0.003	-	-	0.19
ZNP all years	16	0.1	0.005	374	0.0312	0.12
ZNP ewes all years	3	0.08	0.002	-	-	0.42
ZNP 2018	6	0.21	0.003	127	0.639	0.19
ZNP 2019	7	0.19	0.004	292	0.027	0.12
ZNP 2020	8	0.13	0.003	140.5	0.309	0.11
ZNP ewes 2018	3	0.35	0.002	-	-	0.23
ZNP ewes 2019	4	0.1	0.002	-	-	0.07
ZNP ewes 2020	3	0.21	0.002	-	-	0.09

**Figure A1:**
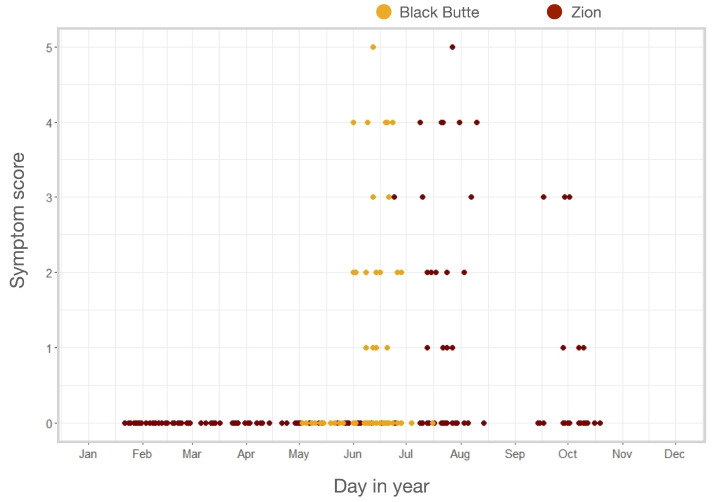
Symptom emergence among lambs in the Zion bighorn population (red) and the Black Butte population (orange), aligned by Julian date.

**Figure A2:**
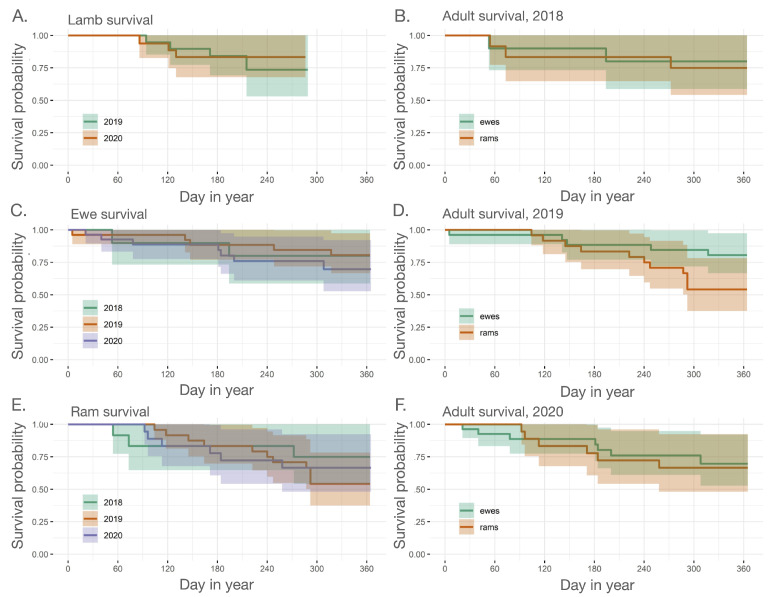
(**A**–**F**) Survival patterns by sex group and year within the Zion population over the duration of this study.

## Data Availability

All data required to reproduce analyses presented in this manuscript will be deposited on Dryad during the revision stage.

## Appendix A

**Table A1:** Adult bighorn mortalities recorded in this study. Subscripts indicate collar deployment numbers.

Animal ID	Subunit	Sex	Age	Date Notified	Cause
$39882_{1}$	ZNP	F	adult	6 January 2019	Avalanche
$39869_{1}$	ZNP	M	adult	29 April 2019	Fall
$42739_{1}$	Barracks	F	adult	22 May 2019	Predation
$39864_{1}$	ZNP	M	adult	26 May 2019	Predation
$42737_{1}$	Barracks	F	adult	28 May 2019	Predation
$39871_{1}$	ZNP	M	adult	14 June 2019	Predation
$42011_{1}$	Barracks	M	adult	1 July 2019	Predation
$39859_{1}$	Hildale	M	adult	29 August 2019	Unknown
$42007_{1}$	Barracks	M	adult	5 September 2019	Trapped
$42738_{1}$	Barracks	F	adult	6 September 2019	Predation
$39878_{1}$	ZNP	M	adult	15 September 2019	Unknown
$39866_{1}$	Hildale	M	adult	20 October 2019	Predation
$39863_{2}$	ZNP	M	adult	20 October 2019	Drowning
19-L3	ZNP	F	lamb	9 January 2020	Uncertain; had pneumonia
$42740_{1}$	Kanab	F	adult	22 January 2020	Predation
$39879_{2}$	ZNP	F	adult	10 February 2020	Predation
$42743_{1}$	Hildale	F	adult	19 March 2020	Fall
$45149_{1}$	ZNP	M	adult	2 April 2020	Predation
$39878_{2}$	ZNP	M	adult	6 April 2020	Predation
$39876_{2}$	ZNP	M	adult	23 April 2020	Predation
$39877_{2}$	Hildale	M	adult	20 June 2020	Predation
$42746_{1}$	Hildale	F	adult	30 June 2020	Predation
$42711_{1}$	Hildale	F	adult	3 July 2020	Predation
$42745_{1}$	Hildale	F	adult	19 July 2020	Predation
$42011_{2}$	ZNP	M	adult	4 November 2020	Starvation
$47815_{1}$	ZNP	F	adult	24 November 2020	Predation
$47177_{1}$	ZNP	F	adult	13 December 2020	Fall
$47454_{1}$	Barracks	M	adult	24 December 2020	Capture myopathy
$39868_{2}$	ZNP	M	adult	9 January 2021	Septicemia
$42006_{1}$	Barracks	M	adult	12 January 2021	Fall

**Table A2:** Lamb fates 2019 and 2020. Subscripts indicate collar deployment number (e.g., 39879${}_{2}$ is the second deployment of collar 39879).

Year	Ewe’s ID	Subunit	Lamb Status	Lamb Fate
2019	$39879_{2}$	ZNP	confirmed 20 June 2019	weaned
2019	$39880_{1}$	ZNP	confirmed 13 May 2019	unknown
2019	$39881_{1}$	ZNP	confirmed 18 July 2019	weaned
2019	$39883_{1}$	ZNP	confirmed 27 February 2019	weaned
2019	$39885_{1}$	ZNP	confirmed 13 March 2019	weaned
2019	$39886_{2}$	ZNP	confirmed 13 March 2019	weaned
2019	$39887_{1}$	ZNP	confirmed 28 May 2019	unknown
2019	$39888_{1}$	ZNP	confirmed fall 2019	weaned
2019	$41992_{1}$	ZNP	confirmed 11 March 2019	drowned 03 August 2019
2019	$41993_{1}$	ZNP	confirmed 28 March 2019	weaned
2019	$41994_{1}$	ZNP	confirmed 28 March 2019	weaned
2019	$41995_{1}$	ZNP	no lamb	-
2019	$42737_{1}$	Barracks	confirmed 30 January 2019	lost by 30 May 2019
2019	$42738_{1}$	Barracks	confirmed 18 March 2019	weaned
2019	$42739_{1}$	Barracks	confirmed 18 March 2019	lost by 26 May 2019
2019	$42740_{1}$	Kanab	confirmed 21 March 2019	weaned
2019	$42741_{1}$	Kanab	confirmed 29 January 2019	lost by 04 April 2019
2019	$42742_{1}$	Barracks	confirmed 11 February 2019	weaned
2019	$42743_{1}$	Hildale	confirmed 19 February 2019	weaned
2019	$42744_{1}$	Barracks	confirmed 9 February 2019	weaned
2019	$42745_{1}$	Hildale	confirmed 22 February 2019	weaned
2019	$42746_{1}$	Hildale	no lamb	-
2020	$42742_{1}$	Barracks	confirmed 8 May 2020	weaned
2020	$42744_{1}$	Barracks	suspected before 22 April 2020	lost by 10 May 2020
2020	$42743_{1}$	Hildale	confirmed 21 February 2020	unknown, ewe died 17 March 2020
2020	$42745_{1}$	Hildale	confirmed 11 April 2020	unknown, ewe died 19 July 2020
2020	$42746_{1}$	Hildale	confirmed 28 May 2020	unknown, ewe died 30 June 2020
2020	$42741_{1}$	Kanab	confirmed 6 February 2020	lost by 31 March 2020
2020	$39880_{1}$	ZNP	confirmed 16 June 2020	weaned
2020	$39881_{1}$	ZNP	confirmed 3 March 2020	weaned
2020	$39882_{2}$	ZNP	confirmed 2 April 2020	weaned
2020	$39883_{1}$	ZNP	confirmed 18 February 2020	weaned
2020	$39884_{1}$	ZNP	confirmed 31 March 2020	weaned
2020	$39885_{1}$	ZNP	confirmed 31 March 2020	weaned
2020	$39886_{2}$	ZNP	confirmed 2 April 2020	weaned
2020	$39887_{1}$	ZNP	confirmed 16 May 2020	weaned
2020	$39888_{1}$	ZNP	confirmed 3 April 2020	weaned
2020	$41992_{1}$	ZNP	confirmed 31 March 2020	weaned
2020	$41993_{1}$	ZNP	confirmed 6 March 2020	lost by 16 May 2020
2020	$42721_{1}$	ZNP	confirmed 3 March 2020	weaned
2020	$42737_{2}$	ZNP	no lamb	-
2020	$42738_{2}$	ZNP	confirmed 19 February 2020	weaned
2020	$42739_{2}$	ZNP	confirmed 15 May 2020	weaned
